# Colloidal Chiral Carbon Dots: An Emerging System for Chiroptical Applications

**DOI:** 10.1002/advs.202305797

**Published:** 2024-01-24

**Authors:** Yuwan Zhao, Juan Xie, Yongzhi Tian, Stefanos Mourdikoudis, Nadesh Fiuza‐Maneiro, Yanli Du, Lakshminarayana Polavarapu, Guangchao Zheng

**Affiliations:** ^1^ School of Physics and Microelectronics Zhengzhou University Zhengzhou 450001 P. R. China; ^2^ Separation and Conversion Technology Flemish Institute for Technological Research (VITO) Boeretang 200 Mol 2400 Belgium; ^3^ CINBIO Materials Chemistry and Physics Group University of Vigo Campus Universitario Marcosende Vigo 36310 Spain; ^4^ Institute of Quantum Materials and Physics Henan Academy of Sciences Zhengzhou 450046 P. R. China

**Keywords:** chiral carbon dots, chiral effect, bio‐ or chemical sensing, drug‐delivery, enzyme‐like catalysis, synthetic strategies

## Abstract

Chiral CDots (c‐CDots) not only inherit those merits from CDots but also exhibit chiral effects in optical, electric, and bio‐properties. Therefore, c‐CDots have received significant interest from a wide range of research communities including chemistry, physics, biology, and device engineers. They have already made decent progress in terms of synthesis, together with the exploration of their optical properties and applications. In this review, the chiroptical properties and chirality origin in extinction circular dichroism (ECD) and circularly polarized luminescence (CPL) of c‐CDots is briefly discussed. Then, the synthetic strategies of c‐CDots is summarized, including one‐pot synthesis, post‐functionalization of CDots with chiral ligands, and assembly of CDots into chiral architectures with soft chiral templates. Afterward, the chiral effects on the applications of c‐CDots are elaborated. Research domains such as drug delivery, bio‐ or chemical sensing, regulation of enzyme‐like catalysis, and others are covered. Finally, the perspective on the challenges associated with the synthetic strategies, understanding the origin of chirality, and potential applications is provided. This review not only discusses the latest developments of c‐CDots but also helps toward a better understanding of the structure‐property relationship along with their respective applications.

## Graphical abstract

Chiral CDots (c‐CDots) not only inherit those merits from CDots, but also exhibit the chiral effects in optical, electric, and bio‐properties. Therefore, significant progress is achieved for c‐CDots as a result of numerous research efforts carried out. The synthetic strategies of c‐CDots, including postfunctionalization of CDots with chiral ligands, and one‐pot synthesis. and assembly of CDots with chiral soft templates. Afterward, a clear chiral effect of c‐CDots on their applications is elaborated.

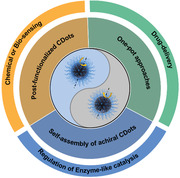

## 1 Introduction

Carbon dots (CDots) or carbonaceous nanoparticles (NPs) belong to a class of 0D carbon material with a size range of a few nanometers to 10 nm.^[^ ^1^ ^]^ These new types of fluorescent nanomaterials were first reported in 2004 after being accidentally discovered following the purification of single‐walled carbon nanotubes synthesized by arc‐discharge methods, displaying bright luminescence and unique crystalline nanostructures.^[^ ^2^ ^]^ CDots have gained increasing attention in the last decade due to their remarkable optical properties, good biocompatibility, low toxicity, and high stability together with the ease of their synthesis procedures. One of the most common synthetic methods for CDots is hydrothermal synthesis assisted with other instruments (i.e., lasers, microwaves, and electrochemistry). CDots generally exhibit quasi‐spherical morphology consisting of sp^2^/sp^3^ carbon atoms and oxygen/nitrogen‐based groups. In addition, their surface consists of various other functional groups, such as hydroxyl, epoxy resin/ether, carbonyl, and carboxyl, making them highly soluble in water or organic solvents, and offering good biocompatibility as well as facile surface functionalization.^[^ ^3^ ^]^ CDots with crystalline graphite cores and organic shells exhibit an absorption peak in the wavelength range of 200–300 nm that is assigned to the π − π* electron transition and another peak in the lower energy region that is related to aromatic *sp* ^2^ C═C bonds of the inherent carbon skeletons. Generally, the chemical motifs of C  =  O,   − NH_2_,  − SH,   − OH,  or C − N bonds induce another absorption peak at a higher wavelength (≈300–400 nm), which is attributed to the n − π* electron transition. The most common surface motifs on CDots are also confirmed by FTIR (**Table** **1**) and XPS (**Table** **2**) spectroscopy. The photoluminescence (PL) properties of CDots are driven by the carbogenic core and surface defects via chemical trapping of excited‐state energy on their surfaces, rather than the band edge emission.^[^ ^4^ ^]^ Along with in‐depth and continuous research on understanding the structure‐property/performance relationship, it has been found that the electronic energy level structure, crystallinity, and surface chemical information on CDots affects their fluorescence properties,^[^ ^5^ ^]^ biocompatibility,^[^ ^6^ ^]^ photochemical stability without bleaching,^[^ ^7^ ^]^ and electron spin polarization. CDots with several promising properties have also been applied in numerous fields, such as devices,^[^ ^8^ ^]^ medicine,^[^ ^9^ ^]^ information encryption,^[^ ^10^ ^]^ bioimaging,^[^ ^11^ ^]^ and catalysis,^[^ ^12^ ^]^ among others.^[^ ^13^ ^]^

**Table 1:** Typical wavenumbers and corresponding vibrational modes in the FTIR spectrum of c‐CDots.

*Wavenumber [cm^−1^]*	Vibrational modes
*3376*	Stretching vibration of N‐H
*3044*	Stretching vibration of O‐H
*2927/2820*	Stretching vibration of C‐H
*2682*	S‐H
*1715*− *1735*	Bending vibration of C=O
*1627*	Bending vibration of C=C
*1591*	C=N
*1490*	C=C
*1410*	‐COO‐
*1133*	C‐N
*1194*	C‐S‐C

**Table 2:** Commonly observed band positions and associated chemical groups in XPS spectra of c‐CDots.

*Peak position (eV*)		Chemical groups
C1s	*284.7*	Graphitic sp2 C=C structure
	*285.5*	C‐O/C‐S/C‐N
	*286.9*	C=N
	*287.2*	O=C‐N/C=O
N1s	*398.5*	pyridinic nitrogen
	*400.64*	pyridinic nitrogen
	*401.62*	N‐H
O1s	*531.85*	C=O
	*532.94*	C‐OH/C‐O‐C
	*533.67*	C‐O
S2p	*163.32, 164.31, 164.65*	C‐S‐C

Recently, numerous studies have suggested the induction of chirality to nanomaterials in order to incorporate new chiroptical properties. Chirality is a geometric property of an object that cannot be superimposed with its mirror image. Their noncentrosymmetric structure confers the chiral materials and their mirror images (enantiomers) interesting optoelectronic properties such as circular dichroism (CD), circularly polarized photoluminescence (CPL), nonlinear optics (NLO), and spintronics. Therefore, chiral materials present relevant applications in many fields such as biology, chemistry, medicine, and spintronic devices.^[^ ^14^ ^]^ Chirality endows the reference body of CDots in the mirror image with distinct physical and chemical properties compared to its own ones, called chiral CDots (c‐CDots) with two enantiomers. In 1998, Robert L. Whetten and his co‐workers reported that chiral gold clusters capped with glutathione peptides exhibited optical activity.^[^ ^15^ ^]^ Chiral nanostructures enable to tune and control the light‐matter interactions in a wide wavelength range. For instance, the chiroptical response of nanomaterials (metals and semiconductors) is tunable from UV–Vis to NIR regions while the CD signal of chiral molecules is usually localized in the UV regions.^[^ ^16^ ^]^

**Scheme 1:**
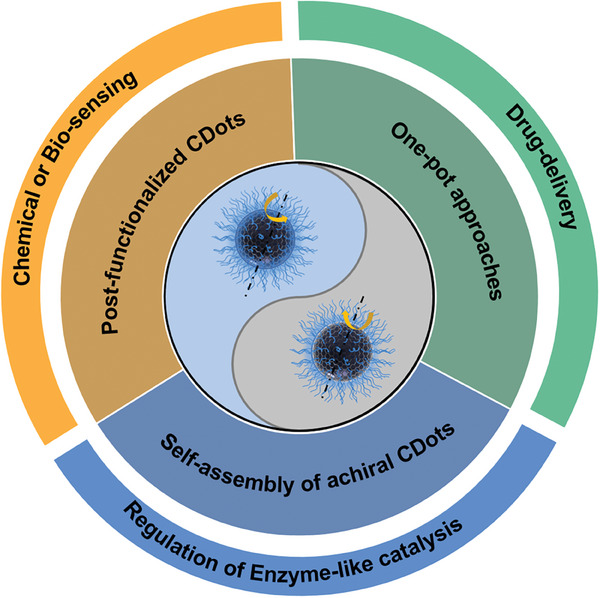
Synthetic strategies of c‐CDots and their chiral effect on applications.

Considering their excellent optical, catalytic, and physiological properties, research on the synthetic methodologies, chirality origins, and applications of colloidal nanocrystals have been significantly explored by several research groups worldwide.^[^ ^17^ ^]^ Different types of nanocrystals including metals, metal oxides, quantum dots (QDs), halide perovskites, metal halide clusters, and c‐Cdots have been found to exhibit chiroptical response under specific conditions (Chiral morphology, chiral ligand functionalization, or chiral self‐assembly).^[^ ^17^ ^]^ Recently, there have been several reviews discussing c‐Cdots, which are mainly focused on synthetic methods along with applications.^[^ ^16^ ^]^ However, the origin of chirality is still not well understood and the structure‐property/function relationship needs to be discussed in detail. Therefore, a detailed review is now timely and worthy to summarize the recent development of c‐Cdots, particularly focusing on the possible origin of chirality and the detailed synthetic strategies and structure‐chiral property relationship and their applications. In this review, we briefly introduce a definition of c‐CDots in section 1, and demonstrate the history of chirality from molecules to nanomaterials, chirality quantifications, chirality origin, and circularly polarized luminescence (CPL) in section 2. Then, we describe the catalog of c‐CDots based on the synthetic methodologies in section 3. Chiral effects on the applications of c‐CDots are subsequently discussed including drug‐delivery, chemical or biosensing, and regulation of enzyme‐like catalysis in section 4. Finally, a brief summary and our prospects are given to conclude the recent advances and the current state of c‐CDots, but also to explain the existing shortcomings that need to be addressed for an even more promising future for such materials in section 5. This review aims to provide a robust frontier for the synthetic methodologies, chirality origin, and applications of CDots with chirality as shown in **Scheme** **1**.

## 2 Chirality Meets Carbon Dots

### 2.1 The Historical Evolution of Chirality in Different Material Systems

**Figure 1:**
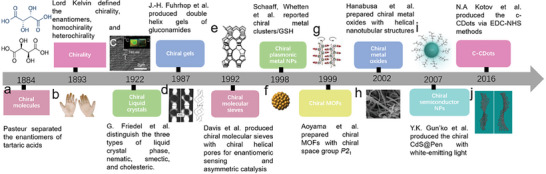
Evolution of chiral matters from molecules to nanomaterials. a) The enantiomeric crystalline structures of tartaric acids were separated by French scientist Pasteur.^[^ ^20^ ^]^ b) The definition of chirality was elaborated by Lord Kelvin.^[^ ^21a^ ^]^ c) SEM images of cholesteric ac‐CDots film with helical pitches.^[^ ^38^ ^]^ Reproduced with permission from ref. [38]. Copyright 2020. American Chemical Society. The nematic, smectic, and cholesteric phases of Liquid crystals were defined by G. Friedel et al.^[^ ^22^ ^]^ d) Electron images of helical gels with their models.^[^ ^23^ ^]^ Reproduced with permission from ref. [23]. Copyright 1987. American Chemical Society. (e) Illustration of enantioenriched chiral molecular sieves.^[^ ^24^ ^]^ Reproduced with permission from ref. [24]. Copyright 1992. American Chemical Society. (f) Chiral gold nanoclusters capped with Gluthathione (GSH) chiral molecules.^[^ ^15^ ^]^ Reproduced with permission from ref. [15]. Copyright 1998. American Chemical Society. g) Schematic of two adjacent helices within the chiral MOFs.^[^ ^26^ ^]^ Reproduced with permission from ref. [26]. Copyright 1999. American Chemical Society. h) SEM images of tantalum oxides helical fiber structures.^[^ ^39^ ^]^ Reproduced with permission from ref. [39]. Copyright 2002. American Chemical Society. i) chiral semiconductor CdS capped with Pen chiral molecules.^[^ ^40^ ^]^ Reproduced with permission from ref. [40]. Copyright 2007. Royal Society of Chemistry. j) The first case of c‐Cdots surface‐functionalized with Cys reported by N.A Kotov et al.^[^ ^32^ ^]^ Reproduced with permission from ref. [32]. Copyright 2016. American Chemical Society.

Chirality plays an important role in physics, chemistry, and pharmacy.^[^ ^17^, ^18^ ^]^ For instance, the birth of children with severe deformities had been attributed to the misuse of the enantiomers of thalidomide, a tranquilizer, in the 1960s.^[^ ^19^ ^]^ The evolution of major advances related to chiral materials that occurred in history is illustrated in **Figure** **1**. Pasteur separated the enantiomers of tartaric acid crystals which contributed to the different rotation degrees of polarized light passing through them. So far, the discipline of stereochemistry and molecular chirality has already arised since long time ago (Figure 1a).^[^ ^20^ ^]^ Sir William Thomson first introduced the chirality concept in science in 1893. Later in the same year, Lord Kelvin defined the chirality, enantiomers, homochirality, or heterochirality as mentioned below: “ *I call any geometrical figure, or group of points, chiral, and say that it has chirality if its image in a plane mirror, ideally realized, cannot be brought to coincide with itself. Two equal and similar right hands are homochirally similar. Equal and similar right and left hands are heterochirally similar. They are also called enantio‐morphs as introduced by German writers I believe. Any chiral object and its image in a plane mirror are heterochirally similar*.”^[^ ^21^ ^]^ With the development of characterization and synthetic methodologies of nanomaterials, chirality emerged at the nanoscale. Some of the most appealing chiral materials include liquid crystals,^[^ ^22^ ^]^ gels,^[^ ^23^ ^]^ molecular sieves,^[^ ^24^ ^]^ noble metal NPs,^[^ ^24^, ^25^ ^]^, metal‐organic frameworks (MOFs),^[^ ^26^ ^]^ metal oxides,^[^ ^27^ ^]^ upconversion NPs,^[^ ^27^, ^28^ ^]^ perovskites,^[^ ^14^, ^29^ ^]^ sulphides^[^ ^30^ ^]^ and CDots^[^ ^31^ ^]^. In 2016, Suzuki et al. reported that chiral graphene quantum dots can be prepared by L‐cysteine or D‐cysteine surface‐functionalization.Based on DFT and semiempirical ZINTO algorithms, the chirality transfer was attributed to the electron transition from molecular scale to graphene quantum dots.^[^ ^32^ ^]^ Since then, c‐CDots, as one important type of chiral nanomaterials, have been developed in the domains of enantiomeric recognition,^[^ ^33^ ^]^ chemical/bio‐sensing,^[^ ^34^ ^]^ chiral catalysis,^[^ ^31^, ^35^ ^]^ drug‐delivery^[^ ^35^, ^36^ ^]^ and optoelectronic devices.^[^ ^36^, ^37^ ^]^

### 2.2 Chirality Quantification

**Figure 2:**
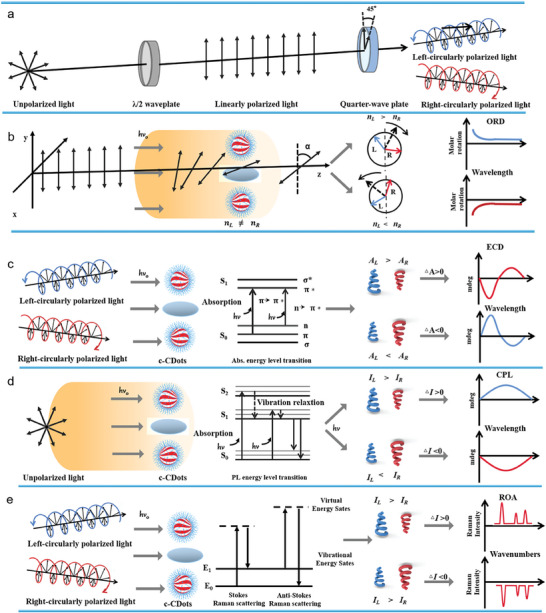
Chirality quantification of c‐Cdots. a) Schematic representation of unpolarised light, linearly polarized light, LCP, and RCP. b) Schematic diagram of ROD based on the different refractive index of chiral materials. c) Schematic diagram of ECD spectrophotometer based on the different electronic absorption of c‐CDots toward LCP and RCP, respectively. d) Schematic diagram of CPL spectrophotometer based on the different fluorescence emission of c‐CDots toward LCP and RCP, respectively. e) Schematic diagram of ROA based on the distinct Raman scattering of chiral motifs on c‐CDots toward LCP and RCP, correspondingly.

One of the promising properties of c‐CDots is the ability to study photo spin‐filtering under the irradiation of Left‐circularly polarized light (LCP) and Right‐circularly polarized light (RCP). Natural light without polarization is regarded as unpolarised light, showing vibrations in all planes. When an unpolarised light passes through the polaroid filter, the light vector vibrates only in a fixed direction in the propagation direction of light, producing linearly polarized light with a fixed vibration plane. By further propagation in the optical path, if the angle between the vibration direction of linearly polarized light and the optical axis of the quarter‐wave plate is +45^o^ or –45^o^, the linearly polarized light emitted from the quarter‐wave plate is LCP and RCP, respectively (**Figure** **2**a).

c‐CDots can display optical rotatory dispersion (ORD), as well as mirror‐like spectra in electronic circular dichroism (ECD),^[^ ^41^ ^]^ circularly polarized dark‐field scattering (CPDFs), Raman optical activity (ROA), vibrational circular dichroism (VCD),^[^ ^42^ ^]^ and circularly polarized photoluminescence (CPL)^[^ ^42^, ^43^ ^]^ or circularly polarized phosphorescence (CPP).^[^ ^38^ ^]^ The optical activity generally arises from the different refractive index (*n_L_* ≠ *n_R_*) of chiral enantiomers, leading to their different propagation speeds of corresponding polarized light. Therefore, ORD is applied to quantify the optical rotation changes at different wavelengths (Figure 2b) due to their distinct refractive indices for right‐ and left‐circularly polarized light.^[^ ^44^ ^]^ On the other hand, the ECD spectrum is obtained according to the extinction (absorption and scattering) difference of c‐CDots under the irradiation of LCP and RCP. When A_L_> A_R_, the ECD spectra have positive values. Otherwise, it bears a negative value. The ECD spectrum often shows a negative and positive cotton effect and the magnitude of the optical rotation crosses zero at absorption maxima (Equation (1) and Figure 2c). The extent of chirality can be quantified by CD spectroscopy measuring the difference in absorption of left or right circularly polarized light respectively.

(1) $$\mathrm{CD}=A_{L}-A_{R}$$

Considering that the absorption depends on the concentration, this magnitude can be normalized according to Equation (2), obtaining the dissymmetry factor or g‐factor. Besides, circularly polarized dark‐field microscopy (CPDFM) is a technique that provides the differential scattering spectroscopy of discrete chiral nanomaterials under the different irradiation of LCP and RCP, revealing the intrinsic and extrinsic chiroptical properties.

(2) $$g_{CD}=\frac{\Delta A}{A}\;=\frac{CD\;\left(mdeg\right)}{Abs\cdot 32980}\;$$

Another relevant quantity is the luminescence dissymmetry factor related to polarized luminescence, which quantifies the ratio of right and left circularly polarized emitted light according to Equation (3). The difference in PL intensity arises from the fluorescence emission spectra of c‐CDots under the excitation of unpolarised light, producing the positive CPL spectra when *I_L_* >  *I_R_*, and negative CPL spectra when *I_L_* <  *I_R_* (Figure 2d).

(3) $$g_{lum}=\;2\frac{\left(I_{\mathrm{LCP}}-I_{\mathrm{RCP}}\right)}{\left(I_{\mathrm{LCP}}+I_{\mathrm{RCP}}\right)}$$

ROA is another effective analytical technique for the structural confirmation of chiral materials based on their different Raman scattering intensities under irradiation of LCP and RCP, respectively (Figure 2e).^[^ ^45^ ^]^ In the 1970s, the principle of ROA was explained by Barron, as follows “the scattered light carries a very small degree of circular polarization and the scattered intensity is slightly different in right‐ and left‐circularly polarized incident light.”^[^ ^46^ ^]^

### 2.3 Origin of Chirality in c‐CDots

Through the characterization of c‐CDots by spectrophotometry measurements and simulations, the chirality origins of c‐CDots are classified into three aspects. i) For discrete c‐CDots, the optical properties originate from the electron transition between the surface motifs and the internal carbon skeletons.^[^ ^46^ ^]^ c‐CDots inherit the helical features of chiral molecules, called “molecular chirality imprints”. The functional chiral motifs on the c‐CDots can retain their own chiroptical performance, which can also endow their chiral property to the Sp2‐hybridized carbon core, resulting in the appearance of a new chiral absorption or a fluorescence emission spectrum. ii) Through carefully controlling the kinetics at the interface of molecules‐crystal facets, the conformation of chiral molecules endows their chirality to nanoparticles, leading to the generation of chiroptical response contributed by the twisted lattice fringes or enantiomeric crystal facets.^[^ ^46^ ^]^ However, it is extremely difficult to determine whether the crystal facets of c‐Cdots are twisted or not because of their extremely small size. iii) randomly or ordered assembly of c‐CDots into chiral supernanostructures, in which the optical properties mainly come from the electron transition between the chiral motifs of neighboring c‐CDots, leading to their chiroptical performance changes.^[^ ^46^ ^]^ For instance, when achiral‐CDots were aligned with the liquid crystal or helical templates, they exhibited chiroptical responses. The helical templates may play the role of circular polarizers. Besides, the twisted morphology also contributes to their chiroptical response.^[^ ^47^ ^]^ Given that most organisms (amino acids, peptides, proteins, DNA, and hands) existing in our bodies are chiral, the unique chiral effect of c‐CDots will provide a promising platform by merit of enantiomeric interaction for the potential bio‐applications, in comparison to achiral CDots. The enantiomers of c‐CDots display different physiological and catalytic properties based on their distinct L‐conformation and D‐conformation, which has to do with the chiral effect of c‐CDots. For instance, c‐CDots derived from L‐lysine have a better chance to regulate the aggregation kinetics, final fiber morphology, and cytotoxicity of Aβ42 (42‐residue‐long amyloid‐β) rather than that derived from D‐lysine, where Aβ42 is one of the pathogenies for Alzheimer's disease.^[^ ^48^ ^]^

### 2.4 Luminescence Properties of c‐CDots

In view of the relevance of the chiroptical properties of the c‐CDots mentioned above, numerous studies have emerged with the aim of obtaining intense circularly polarized photoluminescence reaching high dissymmetry factors, making them excellent candidates for numerous applications such as device performance and sensing among many other.

One of the first approaches carried out for this purpose^[^ ^52^ ^]^ consisted of an arrangement of helical cellulose nanocrystals with CDots, being able to obtain high dissymmetry values of up to −0.74, and being favored by the emission intensity and loading of CDots, as well as the intrinsic helical organization.^[^ ^52^ ^]^ Further studies employed a similar strategy, demonstrating in cell viability tests the non‐cytotoxic character of CDots compounds hybridized with cellulose nanocrystals, suggesting their effectiveness in bioimaging, together with their other applications in targeting and as drug‐delivering agents.^[^ ^53^ ^]^ Moreover, the ability of cellulose NCs to reorganize themselves into 1D photonic crystal lattices enables the fabrication of cholesteric lasers without the presence of mirrors, opening up the possibility of numerous applications.^[^ ^54^ ^]^

**Figure 3:**
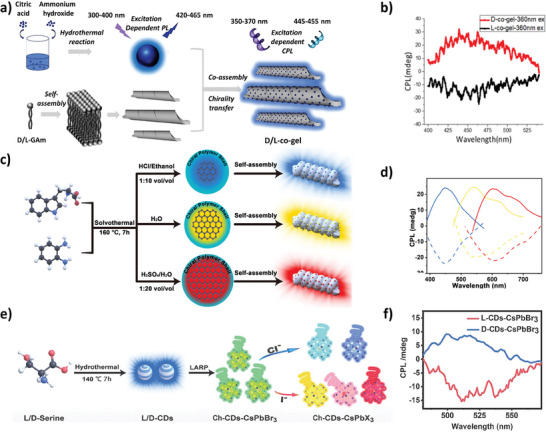
a) Scheme of the formation of the supramolecular co‐assembly of D/L‐co‐gel with excitation‐dependent PL. b) CPL and spectra of D‐co‐gel (red curve) and L‐co‐gel (black curve) with an excitation wavelength of 360 nm.^[^ ^49^ ^]^ Reproduced with permission from ref. [49]. Copyright 2020. American Chemical Society. c) Scheme of the synthetic procedure of multicolor‐emitting c‐CDots. d) CPL spectra of the three chiral carbonized polymer dots co‐gels.^[^ ^50^ ^]^ Reproduced with permission from ref. [50]. Copyright 2021. Wiley‐VCH. e) Scheme of the preparation procedure for c‐CDots‐CsPbX_3_ NCs. f) CPL spectra of c‐CDs‐CsPbBr_3_ NCs.^[^ ^51^ ^]^ Reproduced with permission from ref. [51]. Copyright 2022. Wiley‐VCH.

Subsequently, Zhu et al. developed a new chirality transfer strategy by a super‐assembly through a non‐covalent bonding of nitrogen‐based‐carbon dots with *L*/*D*‐glutamic acid as a chiral‐gelator (**Figure** **3**a–b).^[^ ^49^ ^]^ These co‐assemblies induced excitation‐dependent CPL signals reaching g_lum_ values between 2 × 10^−3^ and 4 × 10^−3^.^[^ ^49^ ^]^ Interestingly, a recent study reported a solvent‐controlled synthetic route for the preparation of excitation‐independent c‐CDots with multicolor and red luminescence.^[^ ^50^ ^]^ The employment of different reaction solvents allows to control of the sp^2^‐conjugated domains and sp^3^‐nonconjugated domains giving rise to emission colors from blue to red (Figure 3c–d).^[^ ^50^ ^]^ Furthermore, the supramolecular self‐assembly of the combination of the organic lipid gel N, N‐bis(octadecyl)‐D/L‐glutamic acid (DGAm) with c‐CDots, gave rise to the multicolor emission of CPL and could generate circularly polarized white light emission with glum values of up to 2.5 × 10^−3^, having potential applications in screens, lighting equipment and probes.^[^ ^50^ ^]^ In the last few years, numerous studies have modulated supramolecular chirality through aggregation forces and solvent‐solvent interactions.^[^ ^55^ ^]^ Recently, an innovative strategy for obtaining CPL in achiral CDots was developed employing two bi‐solvent systems that allowed access to circularly polarized light emission of both handedness.^[^ ^56^ ^]^ Due to the π‐ π intermolecular forces and hydrogen bridges, right and left‐handed nanostructures were obtained by employing different solvent combinations.^[^ ^56^ ^]^

Another novel study was based on the combination of c‐CDots previously synthesized by a hydrothermal strategy with CsPbBr_3_ perovskites by a ligand‐assisted‐reprecipitation method (Figure 3e–f).^[^ ^51^ ^]^ Subsequently, mixed halide perovskites were acquired by an anion exchange process, promoting emission at the entire visible spectra with the purpose of obtaining white light circularly polarized emission. The interaction of the c‐CDots with the CsPbBr_3_ NCs occurs through the COOH and NH_3_ groups of the c‐CDots with the surface metal ions of the perovskite, maintaining the intense luminescence and chirality of the perovskites and chiral carbon dots respectively (g_lum_ = 3.1 × 10^−3^). Interestingly, an inversion of the configuration could be observed in the excited state, which may be due to charge transfer between them.^[^ ^51^ ^]^ As we have summarized, many innovative and emerging studies have developed various strategies in order to generate the highest CPL signals. However, despite the great progress that has been made in recent years, obtaining c‐CDots with high g_lum_ factors remains a field with much potential to be explored further.

## 3 Synthetic Methodologies of c‐CDots

The synthesis routes of c‐CDots has been reported by following numerous methodologies. Prior to concerning the c‐CDots, the synthetic methodologies of achiral CDots can provide guidance for those related to c‐CDots.^[^ ^57^ ^]^ Generally, the manufacturing strategies of achiral CDs are classified into top‐down and bottom‐up approaches. In the former case, achiral CDots are produced by the conversion of carbon materials (i.e., graphite, graphene, carbon nanotubes, carbon black, etc.) into nanoparticles with few nanometers size by means of electrochemical oxidation,^[^ ^58^ ^]^ arc discharge,^[^ ^59^ ^]^ and laser ablation.^[^ ^60^ ^]^ Although the generated CDots show regular morphology, structural integrity, and high crystallinity, chemical compositions on the surface of CDots are difficult to control and modify. In the latter approaches, CDots can be produced by the carbonization of small molecules with carbon skeletons (i.e., carbohydrates, organic acids, and amines). During the carbonization step, the polymerization process takes place to form the cross‐linking polymers. Therefore, the chemical motifs on CDots obtained by the bottom‐up approaches are rich and varied, inheriting the hydroxyl, carboxyl, amino, or other reactive groups from the maternal molecules. In this case, the surface modification of CDots with other functional molecules such as chiral ones will be easier. So, bottom‐up approaches provide a common and convenient route for the production of c‐CDots. In what concerns such bottom‐up pathways, the synthetic methodologies of c‐CDots are classified into three categories, post‐functionalized CDots with chiral ligands, one‐pot approaches, and self‐assembly of CDots with chiral soft templates.

It should be noted that the synthesis of achiral or chiral CDots is usually performed by means of pyrolysis, hydrothermal, microwave, electrolysis, and hydrothermal methods. Pyrolysis is a method that consists of cracking and carbonization of carbon molecules at high temperatures to generate CDots.^[^ ^61^ ^]^ The pyrolysis method is usually carried out at high temperatures, with fast reaction speed, requiring short reaction times. However, the skeletons of synthesized CDots are hard to inherit the structure of carbon molecules. With the hydrothermal methods, the carbonization process takes place in pressurized vessels containing a liquid solvent. The synthetic reactions are elaborated at high temperatures and/or high pressures.^[^ ^35^, ^36^, ^62^ ^]^ Advantages for the CDots produced by this method are simple operation, uniform morphology, and narrow size distribution for the resulting particles, as well as a highly crystalline structure. However, the separation and purification of the product from the reaction solvent is often a challenging task. Microwave‐assisted methods take full advantage of the field energy of microwaves to carbonize the carbon source into CDots.^[^ ^63^ ^]^ During this strategy, there is resonance between the carbon molecules and field energy of microwaves leading to a huge increase of the temperature. This approach can help to reduce energy consumption while achieving a shorter treatment time. However, scale‐up is complicated due to limitations in equipment power. Electrolysis methods are related to the anodic oxidation reaction process of carbon sources.^[^ ^36^, ^64^ ^]^ By varying the voltage, current intensity, and reaction time, carbon sources are oxidized by the electrons to form CDots with different sizes and compositions on the electrodes. The electrolysis methods are conducted under mild conditions. The size and structure of CDots can be well regulated by adjusting the electrolytic time and current which govern their optical and electronic properties. The chemical oxidation method has to do with oxidizing carbon nanorods or fibers into CDots through treatment with strong acids or bases.^[^ ^68^ ^]^ Although this approach does not require complex instruments, the raw materials in this strategy should be carbon nanomaterials in the nanoscale. In addition, the purification and separation process of CDots is cumbersome from harsh solutions.

In the first two methods, CDots mainly acquire chiral structures in two ways. One is to inherit the chirality of the precursor molecule. On the other hand, the generated CDots not only possess a new chiral structure but also maintain the chiral structure of the precursor. Therefore, no matter which synthetic method is used, the amino acid enantiomers, such as tartaric acid (TA), glutamic acid (Glu), tryptophan (Try), lysine (Lys), cysteine (Cys), aspartic acid (Asp) and leucine (Leu), are preferred as raw materials. However, the amino acids are usually employed as a chirality source and a carbon source in a one‐step method. Therein, Cys molecule is favored because it contains both N and S elements, which is often studied as a dopant chirality source. Regarding the third approach, chiral assembly, involves the use of cellulose nanocrystals (CNCs) and chiral gelling agents to construct c‐CDots.^[^ ^69^ ^]^

### 3.1 Postfunctionalized CDots with Chiral Ligands (PFc‐CDots)

In this strategy, pre‐synthesized achiral CDots are necessary to be obtained in the first step via carbonization of active molecules. Afterward, chiral molecules are surface‐functionalized on the surface of achiral CDots through chemical interactions involving covalent bonds, electrostatic interactions, and hydrogen bonds.^[^ ^70^ ^]^ The resulting c‐CDots are named as Lc‐CDots or Dc‐CDots corresponding to the L‐ or D‐ chiral molecules, respectively. In general, carbon molecules, composed of both carbon skeletons and active groups are preferred to be selected as carbon sources of CDots, since the active motifs (i.e., carboxyl, hydroxyl, and amine) on CDots are easier to be surface‐functionalized with chiral molecules. Amino acids are common small molecules in nature, and fundamental elements in life evolution. Therefore, amino acids are normally chosen to be used as chiral domains to endow the achiral CDots with chirality. It should be noted that the surface modification with different chiral molecules will induce dramatically opposite chiroptical responses because the electronic properties and chemical states of CDots are modified. However, the chirality transfer between the chiral ligands and CDots has not been clear up to now and has not been precisely controlled yet. For instance, the solubility of pre‐prepared CDots and chiral ligands varies, leading to the complexity of the post‐surface functionalization process. In addition, the CDots experienced excessive crosslinking or carbonization during this process, leading to the decrease or full elimination of their optical properties.

**Figure 4:**
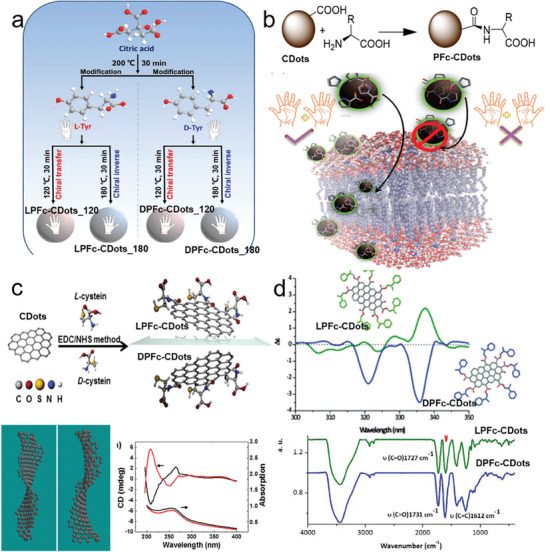
A variety of typical chemical interactions for the PFc‐CDots. a) Schematic diagram of the synthesis of PFc‐CDots via carboxyl groups and active groups of Tyr molecules at different elevated temperatures.^[^ ^65^ ^]^ Reproduced with permission from ref. [65]. Copyright 2021 American Chemical Society. b) Schematics of synthesis of PFc‐CDots via EDC‐NHS methods between cyclic α‐amino acid and CDots.^[^ ^66^ ^]^ Reproduced with permission from ref. [66]. Copyright 2018 American Chemical Society. c) Schematics of synthesis of twisted PFc‐CDots via EDC‐NHS methods between chiral Cys and CDots (upper panel). Theoretical model of twisted PFc‐CDots (left in bottom panel). CD and absorption spectra of twisted PFc‐CDots (right in bottom panel).^[^ ^32^ ^]^ Reproduced with permission from ref. [32]. Copyright 2016 American Chemical Society. d) CD spectra of PFc‐CDots produced from the esterification between chiral phenyl‐1‐propanol and carboxyl group on CDots (upper panel). FT‐IR spectra of LPFc‐CDots (green curves) and DPFc‐CDots (blue curves).^[^ ^67^ ^]^ Reproduced with permission from ref. [67]. Copyright 2016 Royal Society of Chemistry.

There are several chemical or physical interactions for the post‐functionalized CDots with chiral ligands, including the formation of new amide, ester, ‐NH‐CO‐ or hydrogen bonds. Zhang et al. developed one route in which achiral CDots were surface‐functionalized with injected chiral Tyr in a one‐pot approach without a purification process. Achiral CDots were initially obtained by the pyrolysis of citric acid powder. As shown in **Figure** **4**a, by injecting powder of chiral Tyr molecules at designed temperatures, polymerization was initiated on carbon cores between carboxyl groups and active groups of Tyr molecules. The chiroptical response of PFc‐CDots@Tyr strongly depends on the designed temperatures. When the temperature of surface‐functionalization of CDots was at 120 °C, PFc‐CDots@Tyr showed the same handedness as the injected Tyr enantiomer. However, when the above process took place at 180 °C, the handedness of PFc‐CDots behaved in an opposite way. Interestingly, the aromatic structure of chiral amino acids triggered the formation of amide bonds and the conjugation of π − π electrons, contributing to the inverted temperature from 120 to 180 °C (see Figure 4a).^[^ ^65^ ^]^ EDC/NHS method is one way for the cross‐linking of chiral molecules and CDots through injecting an equivalent molar number of EDC and NHS at room temperature. Ostadhossein et al. have prepared the PFc‐CDots via EDC/NHS conjugation of amino acid via carbodiimide coupling on the surface of pristine CDots at mild temperatures. Similarly, these authors also investigated the types of chiral molecules that can induce the different chiroptical behaviors of PFc‐CDots. The only modification of Pro rather than Phe and Cys can lead to the inverted CD signal compared to the CD signal of Pro. The rearrangement of Pro on the surface of CDots during the surface conjugation caused one new orientation of the electronic transition state, resulting in chirality inversion (in Figure 4b).^[^ ^66^ ^]^ Furthermore, the geometry of graphene CDots can be twisted by the surface modification of chiral molecules. The twisted geometry of PFc‐CDots gives rise to distinct optical activities. Suzuki et al. surface‐functionalized graphene CDots with chiral cysteine through EDC/NHS method i) Figure 2c. As a consequence, the resultant PFc‐CDots exhibited a chiroptical response at 210–220 nm assigned to the hybridized molecular orbitals between Cys and edged carbon atoms of CDots, whereas a response at 250–265 nm corresponded to excitonic transition on the twisted geometry of PFc‐CDots. The left and right helicity of PFc‐CDots iii) Figure 4c promote the negative and positive chiroptical response at 250–265 nm.^[^ ^32^ ^]^ Vazquez‐Nakagawa et al. produced PFc‐CDots via the esterification of chiral phenyl‐1‐propanol and the carboxyl group exposed on graphene‐based CDs i) Figure 4d. As shown in ii) of Figure 4d, the generated ester groups were confirmed by the FTIR spectra of C═O stretching vibration modes at 1731−1727 cm^−1^, together with C‐O‐C asymmetric and symmetric stretching vibration modes at 1300 and 1200 cm^−1^, respectively.^[^ ^67^ ^]^

### 3.2 One‐Pot Synthesis of c‐CDots

**Figure 5:**
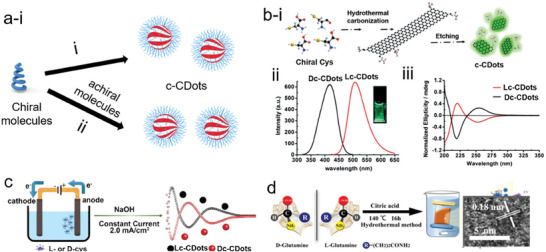
a) Schematic of one‐pot synthesis of c‐CDots via single parent chiral molecules i) and coexistence of chiral molecules and achiral molecules ii). b) Synthesis of c‐CDots via one‐pot hydrothermal carbonization of single Cys, followed by etching process (i). Photoluminescence spectra of Lc‐CDots (ii). iii) CD spectra of Lc‐CDots and Dc‐CDots.^[^ ^71^ ^]^ Reproduced with permission from ref. [71]. Copyright 2018 Wiley‐VCH. c) Synthesis of c‐CDots via one‐pot electrolysis of single Cys with the aid of basic conditions and 2.0 mA cm^−2^ current.^[^ ^72^ ^]^ Reproduced with permission from ref. [72]. Copyright 2018 Royal Society of Chemistry. d) Synthesis of c‐CDots via one‐pot hydrothermal carbonization of Glu and citric acid (left panel). HRTEM images of Lc‐CDots.^[^ ^62b^ ^]^ Reproduced with permission from ref. [62b]. Copyright 2021 Elsevier.

One‐pot approaches help to avoid the time‐spatial separation procedures in comparison to the post‐functionalization methods since post‐surface modification consumes space, time, and energy. On one hand, chiral molecules can be the sole source of carbon skeletons and chiral stimulus, so there is homogenous condensation and carbonization only by the parent chiral molecules i) **Figure** **5**a. On the other hand, chiral molecules play just the role of chiral stimulus, together with other molecules used as the carbon templates, leading to heterogeneous condensation and carbonization processes of chiral stimuli and achiral molecules ii) Figure 5a. Of course, there is no evidence to confirm that not all chiral molecules are used as chiral stimuli. In addition, the kinetics for the condensation between chiral molecules or chiral molecules and achiral carbon precursors should be carefully controlled and tuned for the production of c‐CDots.

Chiral amino acids possessing carbon skeletons and chiroptical properties have been often used for the route (i) of one‐pot approaches. For instance, Nie and co‐workers produced c‐CDots doped with N and S via hydrothermal carbonization methods at 60 °C i) in Figure 5b. Cys not only provided the carbon source of CDots doped with N, S, but also acted as a chiral stimulus endowing its chirality property to c‐CDots. Initially, condensation and carbonization of Cys into big chiral NPs occurred, which were etched with strong basic medium into small c‐CDots. Under the excitation of lasers with 405 nm, 41.2% of PL quantum yield was achieved at 510 nm in Figure 3b‐ii, which is due to the surface trapping and crystalline nanostructures. In the Figure 3b‐iii, there are three typical CD spectra of c‐CDots due to the inherent chiral structure of Cys molecules, π − π* electron transitions and n − π* electron transitions.^[^ ^71^ ^]^ Besides, Hu et al. have used alkaline‐assisted electrolysis methods to produce c‐CDots with a single Cys molecule under a constant current of 2 mA (Figure 5c).^[^ ^72^ ^]^ c‐CDots were produced when Cys underwent a polymerization and carbonization process on the anode electrodes. The chirality origin of CD peaks at 215 nm was due to the inheritance of Cys. In addition, the CD peaks at 248 and 293 nm were attributed to the π − π* conjugation of aromatic Sp2 C═C bonds and spatial configuration of Cys‐syC polymers via S‐S bonds, respectively. During the synthetic reactions, an alkaline medium will be helpful to increase the solubility of Cys with high concentration so that the obtained medium can be used as an electrolyte. With the electrolysis time passing, the CD peaks at 215, 248, and 293 nm appeared, confirming the inheritance, polymerization, and generation of S‐S bonds. It should be noted that electrolysis time is a key parameter in this strategy. A longer reaction time provoked the loss of chiroptical response for c‐CDots. However, in the same research group, when the authors extended this method to Glu‐based c‐CDots, the CD peaks at 205 nm were inverted after 5 days. A red shift of extinction peaks from 208 to 218 nm and a new extinction peak appearing at 283 nm revealed the formation of conjugated aromatic C═C networks and polymerization of Glu.^[^ ^72^ ^]^

In route ii) of one‐pot approaches, chiral molecules together with other carbon sources are simultaneously injected into one vessel, leading to polymerization and carbonization at a suitable temperature. Following this strategy that injected the Gln and citric acid into the reaction vessel, Ma et al. produced c‐CDots with a size of 3.5 nm and [102] facets as determined by the lattice distance of 0.18 nm. The resulting product exhibited chiroptical properties with g_extinction_ values of 0.0001 at 217 nm and 0.00239 at 232 nm. CD peaks of as‐prepared c‐CDots were concentration‐dependent. When the concentration of c‐CDots increased from 0.0675 to 2.0 mg ml^−1^, a red‐shift of CD peaks took place from 209 to 232 nm because of an electronic transition between neighboring carbonyl groups on the aggregated nanoparticles. Furthermore, all the CD signals at lower wavelengths than 250 nm indicated that the chirality origin was coming from neighboring carbonyl groups based on the quantum chemistry simulations (Figure 5d).^[^ ^62b^ ^]^

### 3.3 Self‐Assembly of Achiral CDots into c‐CDots

The assembly of chiral molecules into organisms is a spontaneous phenomenon in nature.^[^ ^73^ ^]^ Inspired by nature, many nanomaterials are self‐assembled into chiral nanostructures stimulated by chiral stimuli (e.g., amino acids, DNA, proteins, chiral surfactants, circularly polarized light, etc). Following this strategy, most achiral Dots can be assembled with chiral soft templates for the production of assembled c‐CDots (ac‐CDots) displaying enhanced chiroptical properties in comparison to the one‐pot methods and PFc‐CDots. However, the whole process should be carefully controlled by adjusting the pH, electrostatic interaction, and the concentration of ions, among other factors. In addition, the chiroptical properties of ac‐CDots are not stable nor durable since they are vulnerable to environmental conditions, i.e. humidity and temperature.

**Figure 6:**
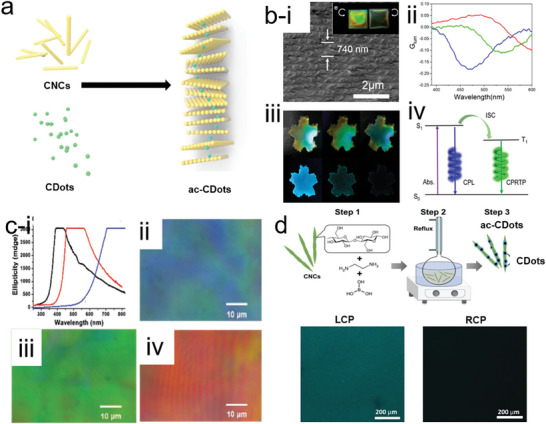
a) Construction strategies of ac‐CDots via self‐assembly strategies. b) i) SEM images of cholesteric ac‐CDots film with helical pitch 740 nm. The inset images in (i) were the photos of the ac‐CDots film illumination under LCP and RCP, respectively. ii) g_lum_ value of c‐CDots films with different helical pitch (Blue: 420 nm; Green: 600 nm and red: 740 nm). iii) Photography of ac‐CDots film illumination under natural light, LCP, and RCP, respectively (from left to right in the upper panel); The ac‐CDots films were excited by with the 254 nm lasers, photoluminescence images of the films were recorded during turning off the lasers 2 s from left to right ((ii) in the bottom panel). (iv) The proposed CPRTP mechanism for the ac‐CDots film.^[^ ^38^ ^]^ Reproduced with permission from ref. [38]. Copyright 2020 American Chemical Society. c) (i) Chiroptical characterization of ac‐CDots/CNCs (black curve: emission blue light; red curve: emission green dots and blue curve: red dots). Polarized optical microscopy (POM) images of ac‐CDots/CNCs (blue), ac‐CDots/CNCs (green), and ac‐CDots/CNCs (red).^[^ ^90^ ^]^ Reproduced with permission from ref. [90]. Copyright 2018 Wiley‐VCH. d) ac‐CDOts produced on the discrete CNCs (upper panel). The confocal fluorescence microscopy images of ac‐CDots/CNCs solution under the irradiation of LCP and RCP (bottom panel).^[^ ^69^ ^]^ Reproduced with permission from ref. [69]. Copyright 2019 Wiley‐VCH.

CNCs, used as one chiral soft‐template, can induce the self‐assembly of nanomaterials into cholesteric films via simple liquid evaporation‐induce methods. In particular, the presynthesized achiral CDots are mixed with CNCs homogeneously, by being gently placed at a suitable temperature and humid environment. After several days, the hybrid assembled c‐CDots (ac‐CDots) cholesteric film composed of achiral CDots and CNCs exhibit excellent chiroptical properties (**Figure** **6**). In most cases, the prepared achiral CDots possess electronegative charges. This will stabilize the negatively charged CNC gels during the assembly routes, thus avoiding the loss of performance by agglomeration. Strictly, this method can also be classified into postfunctionalized strategy since the achiral CDots should be prepared before the assembly process. However, in this review, we have stressed it in a separate section because the 3D helical geometries of assembled nanostructures contribute to the chirality origin (**Table** **3**).

**Table 3:** Synthetic strategies, chiral stimulus, g‐factor, and applications of c‐Cdots.

Synthetic strategies		Carbon source	Chiral stimulus	g‐factor×10^−3^	Applications/REF
One‐pot approaches	Microwave methods	benzoquinone	Cys	–	Moisture sensing ^[^ ^71^ ^]^
	Electrolysis	Cys	Cys		Enzyme catalysis^[^ ^72^ ^]^
	Electrolysis	Glu	Glu	–	Inhibited enzyme activity^[^ ^74^ ^]^
	Hydrothermal	Cys	Cys	–	Cellular metabolism^[^ ^34a^ ^]^
		Cys	Cys	–	Drug delivery^[^ ^75^ ^]^
		Glucose	Glucose	–	Electrocatalysis^[^ ^76^ ^]^
	Hydrothermal	Citric acid	Gln	0.1 or 2.39	Chemical sensing^[^ ^62b^ ^]^
			His	–	
			Arg	–	
		Citric acid	Cys	–	Bioimaging ^[^ ^77a^ ^]^
		Lys	Lys	–	Drug delivery ^[^ ^48^, ^78^ ^]^
		Citric acids	Glu	–	Antibacterial^[^ ^77b^ ^]^
		Citric acids	Cys	–	Plant growth^[^ ^62a^ ^]^
		Citric acid	Asp	–	Sn^2+^ and L‐lys sensing^[^ ^79^ ^]^
		Citric acid	Glu	–	Cancer therapy^[^ ^105^ ^]^
		o‐ phenylenediamine	Tyr	–	Detection of water ^[^ ^34b^ ^]^
		vine tea	Natural deep eutectic solvents	–	Chemical sensing ^[^ ^80^ ^]^
		Citric acid	cyclohexane diamine	–	Chiral‐selected electron transfer^[^ ^81^ ^]^
		N‐methyl‐1,2‐benzenediamine dihydrochloride	L‐Trp	–	Enantiomeric discrimination^[^ ^82^ ^]^
		Citric acid	Cys, Thr or GSH	–	Chiral light^[^ ^83^ ^]^
		Citric acid	Cys	–	Ile sensing^[^ ^84^ ^]^
Postfunctionalized CDs with chiral ligands	Amide bonds	Citric acids	Tyr	1	Electrochemical glucose sensor^[^ ^65^ ^]^
	EDC/NHS	Sucrose	Pro, Phe, or Cys	–	Cell viability^[^ ^66^ ^]^
	EDC/NHS	Graphene sheet	Cys	0.1	Biological activity^[^ ^32^ ^]^
	esterification	graphite	2‐phenyl‐1‐propanol	–	—^[^ ^67^ ^]^
		Citric acid, ethylenediamine	L‐Cys	–	Enantiomeric discrimination^[^ ^85^ ^]^
	—	Cane molasses	Cys	–	Ion sensing^[^ ^86^ ^]^
Self‐assembly methods	CDots		CNCs	g_lum_ = ‐0.27; g_RTP_ = ‐0.47;	—^[^ ^38^ ^]^
	CDots		CNCs	g_lum_ = ‐0.2;	bioimaging^[^ ^69^ ^]^
	CDots		chiral helical polyacetylene	g_lum_ = 0.14 g_RTP_ = 0.012	Imaging^[^ ^87^ ^]^
	CDots		Chiral solvent limonene	–	Imaging^[^ ^88^ ^]^
	CDots		cholesteric liquid crystal	g_RTP_ = 1.09	circularly polarized room‐temperature phosphorescent imaging^[^ ^89^ ^]^

Tyrosine: Pro: Proline; Phe: phenylalanine; Cys: Cysteine; Gln: Glutamine; His: Histidine; Arg: Arginine; Glu: glutamic acid; lum: Luminescence; RTP: room‐temperature phosphorescence; Asp: L‐aspartic acid; Trp: tryptotophan; Thr: Threonine; GSH: Glutathione

Regarding the liquid evaporation‐induced self‐assembly methods, Xu et al. mixed CNCs, Cdots, and poly(vinyl alcohol) (PVA) together for the production of chiral photonic films, which is ac‐CDots. The helical pitch of chiral hybrid films was varied from 420 to 740 nm regulated by the CNCs/PVA ratio. The helical pitch was clearly shown at ≈740 nm, as well as a different cholesteric color appeared under the irradiation of LCP and RCP (i) in Figure 6b). The c‐CDots films presented varying CPL properties as a function of their different helical pitch (ii) in Figure 5b). Furthermore, PVA can stabilize the triplet excitons extending the lifetimes to 103 ms with phosphorescence asymmetric factors of –0.47. The ice‐blue color of ac‐CDots film degraded upon turning off 254 nm laser excitation, enabling to maintenance of the color for 2s (iii) in Figure 6b).^[^ ^38^ ^]^ Due to the photonic bandgap of CNCs, it provides the chiral environment for favoring circularly polarized luminescence and phosphorescence of the hybrid cholesteric film (iv) in Figure 6b). Zheng et al. used the evaporation‐induced self‐assembly strategy to add CDots of blue, green, red, and all three colors to the suspension of CNCs (i) in Figure 6c). After carrying out ultrasonic treatment and evaporation processes, a chiral nematic film was formed showing strong circularly polarized luminescence. The rainbow color pictures of ac‐CDots/CNC film captured by the camera are depicted in Figure 5c‐ii to iv, which is the result of selective reflection of left‐handed circularly polarized light under natural light. On the other hand, the mixture of CNCs and carbon precursors was heated in a one‐pot stage, leading to the assembly of the generated CDots onto the rod‐like CNCs templates.^[^ ^90^ ^]^ Kumacheva and colleagues have produced N‐doped ac‐CDots via injection of ethylenediamine, boric acid, and CNCs into one reflux pot, in which boric acids were used as catalysts to promote the polymerization of ethylenediamine and glucose domains on CNCs (Figure 6d). The size of CDots became larger when the reaction time was extended from 3 to 21 h. The emitted light at 460 nm was resolved by the circular polarizers, showing that the intensity of LCPL was ≈12% higher than RCPL. Furthermore, ac‐CDots display a clearer bright image under LCP than under RCP, revealing the LCPL emission priority (bottom panel in Figure 6d).^[^ ^69^ ^]^

## 4 Chiral Effect on the Applications of c‐CDots

CDots with numerous properties have been widely applied in the fields of drug delivery, chemical or bio‐ sensing, antibacterial activities, catalysis, and other domains. Those applications can be extended by the specific functional motifs of c‐CDots when CDots meet with chirality. In particular, the distinct applications of c‐CDots strongly rely on their L‐configurations or D‐configurations, defined as a chiral effect. In this review, we have focused on the most recent applications based on the chiral effect of c‐CDots.

### 4.1 Drug‐Delivery

Thanks to their excellent biocompatibility and low cytotoxicity, CDots can be widely used in various biomedical applications, such as cell imaging, transformation of cell structure, inhibition of cytotoxicity, antibacterial uses, and others. Several research endeavors have concluded that the c‐CDots present chiral effects on their applications.

**Figure 7:**
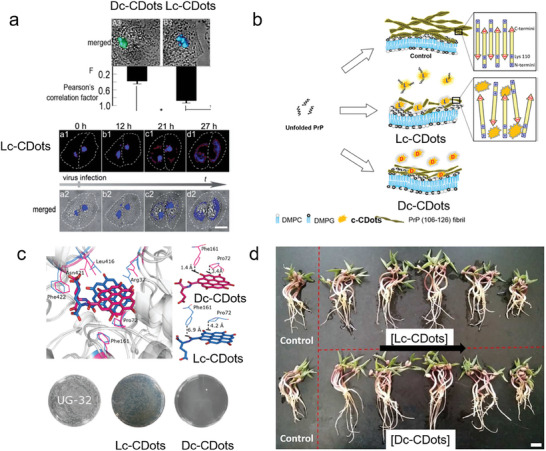
Drug delivery of c‐CDots. a) Merged fluorescence and bright field images of c‐CDots and Pearson correlation factor between Golgi GFP and CQD calculated using Image Pro Plus 6.0. Fluorescence image of Golgi apparatus bodies of HEp‐2 cells stained with LC CQD.^[^ ^77a^ ^]^ Reproduced with permission from ref. [77a]. Copyright 2017. Royal Society of Chemistry. b) Proposed model for the effect of Lc_CDots on the inhibition of chiral PrP fibril.^[^ ^48^ ^]^ Reproduced with permission from ref. [48]. Copyright 2018 Wiley‐VCH. (c) The different total binding free energy was calculated from the Dc‐CDots (hot pink sticks) and Lc‐CDots (blue sticks) (upper panel). Bottom panel: Antimicrobial activities of UG‐32, Dc‐CDots, and Lc‐CDots against E.coli shown by a standard plate count method.^[^ ^77b^ ^]^ Reproduced with permission from ref. [77b]. Copyright 2017 Wiley‐VCH. (d) Growth photography of mung bean treated with the Lc‐CDots and Dc‐CDots as the concentration increased from 0, 10, 50, 100, 500 to 1000 µg mL^−1^ during 5 days with natural light, respectively.^[^ ^62a^ ^]^ Reproduced with permission from ref. [62a]. Copyright 2018 Royal Society of Chemistry.

For instance, the Golgi apparatus is an important platform for protein sorting, processing, and specific transportation. Li et al. observed that Lc‐CDots possessed around two times stronger Golgi targeting ability than Dc‐CDots. The L‐type stereo structure of Lc‐CDots based on Pearson's correlation factor dominates this priority, although the cysteine motifs on both Lc‐CDots and Dc‐CDots render them preferentially positioned on the Golgi apparatus. The photostability and long‐term targeting ability of Lc‐CDots have been applied to in‐situ monitor the morphology of the Golgi apparatus as it collapsed after being treated with virus, as shown in **Figure** **7**a.^[^ ^77a^ ^]^

c‐CDots have been investigated to be used as a drug to inhibit protein aggregation, especially Prp, Aβ42, and other amyloid proteins.^[^ ^91^ ^]^ The c‐CDots synthesized via hydrothermal synthesis of Lys enantiomers reported by Arad et al. were applied to inhibit the prion peptides (PrP (106–126)) in the presence of DMPC:DMPG lipid bilayers. In contrast, PrP(106–126) preferred to undergo a fibrillation pathway on the lipid bilayers. From the shapes, lengths, and amount of fibrils in Figure 7b, the fibrils evolved into short, non‐twisted structures with a decreased amount in the presence of c‐CDots. This clearly shows that c‐CDots behave as good amyloid inhibitors. In particular, Lc‐CDots displayed better modulation than that exhibited by Dc‐CDots on the structural transformations and aggregation of PrP (106–126). Lys residues on the c‐CDots interacted with carboxyl termini (nearby residue 126), and inhibited the assembly of β‐sheets, thus causing a chiral effect on the amyloid inhibitors.^[^ ^48^ ^]^ The same researchers also used the c‐CDots generated from Lys as the chiral drug to modulate the aggregation and cytotoxicity of Aβ42. Similarly, Lc‐CDots have also chiral effects on the inhibition of transformation from random coil to β sheets, while Dc‐CDots almost did not break the network of Aβ42 fibrils. As a result, the cell cytotoxicity of Aβ42 fibrils treated with Lc‐CDots was nearly negligible in comparison to that treated with Dc‐CDots decreasing the side effects of Aβ42 fibrils on cells.^[^ ^48^ ^]^

In comparison to other antibiotics, CDots show promising antimicrobial activity, inducing bacterial resistance at a minimum degree. Furthermore, optional toxicity toward bacterial rather than mammalian cells is also one evaluation criterion for the antibacterial performance. CDots with abundant chemical motifs provide one excellent platform for optional toxicity. Xin et al. have produced Dc‐CDots with D‐Glu motifs, which passed into the bacterial cells and destructed the cell walls by selective interaction with cytoplasmic MurD (upper panel in Figure 7c). Therefore, the bacteria were killed by Dc‐CDots antibacterial agents. However, Lc‐CDots with L‐Glu loses this effect on the antibacterial killing owing to the stereochemical selection of MurD ligase toward D‐Glu substrates prohibiting the synthesis of peptidoglycan. The authors found that the 5–32 µg mL^−1^ of Dc‐CDots made the E. coli population decrease from 68% to 99.9%, in comparison to the negligible antibacterial effect of Lc‐CDots (bottom panel in Figure 7c).^[^ ^77b^ ^]^

Because of their good biocompatibility, water solubility, and easy modification, CDots are easy to be absorbed by plants and show low toxicity to agricultural fertilizers. Due to the chiral effect, the usage of c‐CDots will show a positive or negligible effect on plant growth. C‐Cdots were prepared by a one‐pot hydrothermal treatment of citric acids and Cys. Zhang et al. have investigated the effect of those c‐CDots on the growth of mung bean plants. At the optimal concentration (100 µg ml^−1^) of c‐CDots, both Dc‐CDots and Lc‐CDots have promoted the root activity and Rubisco enzyme activity of bean sprouts (Figure 7d). However, the increased amount of Dc‐CDots was 28.9% and 67.5%, respectively, which were three times higher than that of Lc‐CDots. This chiral effect was mainly contributed by the stronger ability of Dc‐CDots to increase the photosynthesis and accumulation of carbohydrates in mung beans.^[^ ^62a^ ^]^

### 4.2 Chemical or Bio‐Sensing

Based on the polarized‐electron excitonic transition and luminescence properties, the change in the intensities or positions of CD or PL spectra of c‐CDots treated with the targeted analytes can quantitatively and qualitatively reveal the concentration of chemicals or biological compounds. Besides, the enantioselective interactions of c‐CDots with the chiral analytes illustrate that the specific handedness of analytes is based on the chirality‐dependent changes in the profiles of CD or PL spectra.

Most of the amino acids in nature have enantiomers, but only the amino acids with L‐configuration can be absorbed by the human body and play a positive role in healthy conditions. However, those with D‐configuration are toxic to the human body or cause metabolic abnormalities.^[^ ^92^ ^]^ Amino acids are usually added to food species to facilitate human access, and it is easy to cause racemization of amino acids in the process of addition, which will drive the occurrence of side effects in the human body.^[^ ^92^, ^93^ ^]^ The identification and differentiation of chiral drugs are normally very important because often only one medicine can play a normal pharmacological role in the human body, while another may become useless or even harm the human body.^[^ ^94^ ^]^ For example, the drug dopamine is used to treat Parkinson's disease and only levodopa has therapeutic activity, while the accumulation of D‐dopamine in the body will cause harm.^[^ ^95^ ^]^ Regarding the commonly used drug Penicillamine, its S (‐) – enantiomer has immunosuppressive and anti‐rheumatic effects; on the other hand, its R (+) enantiomer can provoke the onset ofcancer.^[^ ^96^ ^]^ Therefore, enantiomeric recognition of amino acids or medicines is crucial. CDots have unique fluorescence characteristics. So far, chromatography is usually applied for chiral recognition and separation. However, the time consumption, high cost, and low accuracy restrict their application. The chemical or biosensors based on the c‐CDots using the CD spectra, absorption spectra, luminescence spectra, or CPL spectra constitute one quite convenient and sensitive sensing nanotechnology with fast response and simple operation.

**Figure 8:**
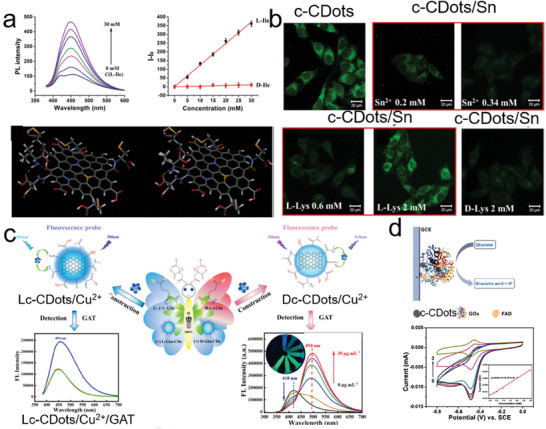
Chemical or biosensing based on the c‐CDots. a) The intensity of PL spectra changes as the concentration of L‐Ile increases (right images in the upper panel). The linear relationship between the increase in PL intensity and the concentrations of L‐Ile (black dots) and D‐Ile (red dots) (left images in the upper panel). Molecular dynamic simulation for the interaction of chiral Ile molecules (right one: D‐Ile; left one: L‐Ile) and Lc‐CDots (bottom panel).^[^ ^84^ ^]^ Reproduced with permission from ref. [84]. Copyright 2021 Elsevier. b) Confocal microscopy images of L929 cells treated with c‐CDots (0.1 mg mL^−1^) (null border image in upper panel) followed by the addition of Sn^2+^ (red border image in the upper panel). b) Confocal microscopy images of L929 cells treated with c‐CDots/Sn followed by the addition of L‐cys (red border image in the bottom panel) and D‐Cys (null border image in bottom panel).^[^ ^79^ ^]^ Reproduced with permission from ref. [79]. Copyright 2020 Elsevier. c) The protocols for the fluorescence sensing of Cu^2+^ ions with the Lc‐CDots and Lc‐CDots, respectively.^[^ ^97^ ^]^ Reproduced with permission from ref. [97]. Copyright 2022 Elsevier. d) Schematics of glucose sensors based on the c‐CDots/GOx (upper panel). CV curves of c‐CDots/GOx in the different glucose concentrations (bottom panels).^[^ ^65^ ^]^ Reproduced with permission from ref. [65]. Copyright 2021 American Chemical Society.

L‐Ile can control blood sugar, repair muscles, and supply energy for physical activities.^[^ ^98^ ^]^ Hou et al. synthesized blue‐emitting Lc‐CDots by hydrothermal method using L‐cysteine and citric acid. The fluorescence intensity at 456 nm of Lc‐CDots was significantly increased after the treatment with L‐Ile (image on the left in the upper panel of **Figure** **8**a), but the luminescence intensity was kept almost constant when treated with D‐Ile. Hou et al. have built the corresponding relationship between Lc‐CDots with the concentration of L‐Ile and D‐Ile, as shown in Figure 8a. The good linear relationship equation clearly revealed that the LOD of L‐Ile is 0.29 mM (left image in the upper panel of Figure 8a). The enantiomeric sensing of L‐Ile with Lc‐CDots is due to the stereoscopic interaction of the π‐donor hydrogen bond and carbon‐hydrogen bond of L‐Ile with the L‐cys motifs and aromatic internal structure of Lc‐CDots. Meanwhile, there is only hydrogen bond interaction between D‐Ile and L‐cys motifs on Lc‐CDots (bottom panel in Figure 8a).^[^ ^84^ ^]^ L‐lysine (L‐Lys) plays an important role in maintaining normal human normal activities, promoting human growth and development, and repairing nerve cells.^[^ ^99^ ^]^ However, a side‐effect disease was caused by the abnormal metabolism of L‐Lys.^[^ ^100^ ^]^ Zheng and his colleagues have developed one simple and appropriate strategy for the detection of Lys enantiomers, as well as for the detection of Sn^2+^. Initially, they synthesized c‐CDots by one‐step hydrothermal method using citric acid as the carbon source and aspartic acid (L‐Asp) as the chiral source. In the presence of Sn^2+^, the intensity of luminescence spectra was decreased due to the coordination between Sn^2+^ and c‐CDots, which behaves as concentration‐dependent. The limitation of detection of Sn^2+^ was as low as 0.057 µM. The fluorescence quenching from the confocal microscopy images of L929 cells occurred with the injection of 10 mM of Sn^2+^. Upon the injection of L‐Lys, the strong coordination ability of L‐lys with Sn^2+^ made the Sn^2+^ detach from the surface of c‐CDots, leading to fluorescence recovery. However, the weak coordination ability of D‐Lys with Sn^2+^ could not drive the Sn^2+^ away from the surface of c‐CDots (Figure 8b).^[^ ^79^ ^]^

Gatifloxacin (GAT) is an important antibiotic and is commonly used to treat bacterial infections.^[^ ^101^ ^]^ However, due to slow metabolism, GAT can accumulate in biological objects and endanger the human body.^[^ ^102^ ^]^ Its residues will also enter the environment through the food chain, which may enhance the drug resistance of microorganisms and cause them to propagate wantonly.^[^ ^103^ ^]^ Chen and co‐workers have developed one chiral fluorescent probe based on c‐CDots (Glu) with Cu^2+^ for the detection of GAT. As shown in Figure 8c, the addition of Cu^2+^ caused fluorescence quenching of c‐CDots (Glu) for the preparation of chiral probes c‐CDots (Glu)/Cu^2+^. Once injected the GAT into Dc‐CDots (Glu)/Cu^2+^, the fluorescence peak at 418 nm decreased as well as the emission peak at 494 nm rose according to the concentration of GAT, while the LOD of GAT was ≈0.04995 µg mL^−1^. However, there was no change in the fluorescence spectra apart from the case of Lc‐CDots (Glu)/Cu^2+^ probes (Figure 8c). Excessive Fe^3+^ will cause environmental pollution and adversely affect the survival of animals and plants. Therefore, a simple and effective method is needed for the sensing of Fe^3+^ in the environment. The as‐prepared c‐CDots (Gln) were used to selectively detect the Fe^3+^ ions in solution via fluorescence quenching due to the electron transition from c‐CDots to Fe^3+^. As the concentration of Fe^3+^ increased, the PL spectra intensity of c‐CDots (Gln) was quenched by 80%, revealing that the lowest detected concentration of Fe^3+^ was ≈0.014 mmol L^−1^.^[^ ^97^ ^]^ Zhang et al. also found that the secondary structure of glucose oxidase was governed by the c‐CDots based on the CD peak intensity of α‐helix and increasing β‐helices at 208 and 218 nm, respectively. In addition, when the hybrids of c‐CDots/glucose oxidase were fabricated for the electrocatalysis of flavin adenine dinucleotide (FAD), c‐CDots would also increase the electron transfer to glucose oxidase. The hybrid electrocatalysts can catalyze glucose into gluconic acid as well as carry an oxidation‐reduction reversible process of FAD. The peak current at the anode was decreased linearly versus the concentration of glucose which ranged from 0.25‐3 (Figure 8d).^[^ ^65^ ^]^

### 4.3 Regulation of Enzyme‐Like Catalysis

c‐CDots not only have good biocompatibility and biodegradability but also have good enantioselectivity to target molecules with specific configurations. Therefore, the addition of c‐CDots can promote and govern the catalytic and enantioselective properties of enzymes.

**Figure 9:**
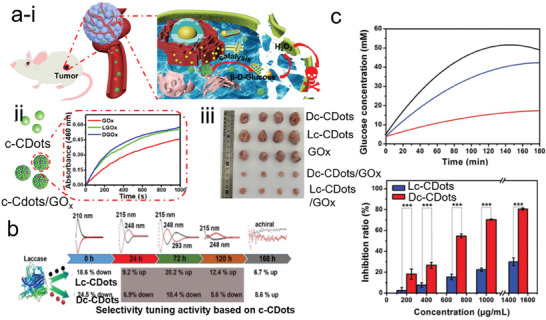
a) i) Schematics of cancer therapy based on the regulation of H_2_O_2_ enzyme catalysis. ii) The absorbance of O‐dianisidine at 460 nm was recorded after the treatment with the GOx (red curve), Lc‐CDots/GOx (Green curve), and Dc‐CDots (blue curve). iii) Representative tumor images of mice after undergoing different treatments with nano drugs.^[^ ^105^ ^]^ Reproduced with permission from ref. [105]. Copyright 2021. American Chemical Society. b) Schematic of catalytic performance of laccase after being treated with the different types of Lc‐CDots and Dc‐CDots.^[^ ^72^ ^]^ Reproduced with permission from ref. [72]. Copyright 2018 Royal Society of Chemistry. c) The glucose concentrations were monitored at different hydrolysis times treated with maltase (black line), maltase/Lc‐CDots (blue line), and maltase/Dc‐CDots (red line), respectively (upper panel). The bar chart of the inhibition ratio with the different concentrations of maltase/Lc‐CDots (blue bar) and maltase/Dc‐CDots (red bar), respectively (bottom panel).^[^ ^74^ ^]^ Reproduced with permission from ref. [74]. Copyright 2019 Wiley‐VCH.

D‐glucose is widely present in the living body. The D‐glucose treated with D‐glucose oxidase enzyme can produce gluconic acid and hydrogen peroxide which displays cytotoxicity for cancer cells and tumors.^[^ ^104^ ^]^ To make good utilization of D‐glucose, CDots used as cargo normally carried glucose oxidase together for the catalytic performance regulation of glucose oxidase. The stability, efficient delivery, and selectivity of glucose oxidase for cell permeability are modified. Zheng et al. opted for the c‐CDots as co‐catalysts loaded with the glucose oxidase for cancer therapy, in which Lc‐CDots/glucose oxidase and Dc‐CDots/glucose oxidase were prepared by the coassembly of glucose oxidase with Lc‐CDots and Dc‐CDots, respectively. It was demonstrated that the generation efficiency of intracellular H_2_O_2_ was enhanced for cancer therapy in the microenvironment, due to the fact that the cellular uptake of glucose oxidase was improved under the cooperative action of c‐CDots (i) in **Figure** **9**a). The increased relative performance of Lc‐CDots/glucose oxidase (51.21%) toward the enzymatic reaction of horseradish peroxide with O‐dianisidine was lower than that of Dc‐CDots/glucose oxidase (60.55%) (ii) in Figure 9a). After 6 days of treatment with PBS solution, the catalytic performance of Dc‐CDots/glucose oxidase was still enhanced while those of Lc‐CDots/glucose oxidase and pure glucose oxidase decreased. The enhanced stability and enzymatic activity by Dc‐CDots was due to the high‐affinity capacity toward glucose. In addition, the Dc‐CDots made the secondary structure of glucose oxidase loose by decreasing the quantities of α‐helix and increasing the quantities of β‐strand. Tumor‐bearing mice experiments revealed that the Dc‐CDots/glucose oxidase was the most efficient additive to restrain tumor growth because the highest intracellular H_2_O_2_ was generated in the Dc‐CDots/glucose oxidase treated tumor (iii) in Figure 9a).^[^ ^105^ ^]^

In addition, c‐CDots play a vital role as catalytic promoters or inhibitors when co‐existing with other catalysts because of their abundant surface chemical motifs. The c‐CDots (Cys) were investigated to regulate the laccase activity toward the oxidization of ABTS substrates, showing a clear chiral effect (Figure 9b). The usage of 0.075 mg mL^−1^ of Lc‐CDots (Cys) benefitted the laccase activity so the increase in catalytic performance was up to 20.2%. This positive effect of Lc‐CDots (Cys) was mainly ascribed to the decrease of the ratio of the secondary structure (β sheet and β turn content). However, Dc‐CDots (Cys) with the same amount inhibited the laccase activity by 10.4% because of the increased β sheet and β turn content which made the laccase compact secondary structure. Initially, the optical properties of c‐CDots (Cys) were tuned by the time period of electrolysis which is related to surface chemical states. As shown in Figure 9b, the alterations in the catalytic activity also strongly relied on their nanosize and surface chemical states.^[^ ^72^ ^]^ In contrast, Kang and colleagues applied the secondary structure of maltase mediated by c‐CDots to inhibit the hydrolytic rate of maltose, decreasing the blood glucose level (Figure 9c). The c‐CDots/maltase were combined by the noncovalent bonds. The higher CD intensity of Lc‐CDots/maltase at 208 and 218 nm than that of Dc‐CDots/maltase, revealed a slight change of secondary structure by the treatment of Dc‐CDots. Meanwhile, c‐CDots in maltase isolated the enzymatic active sites from the substrates. The occupied active sites by c‐CDots decreased the enzymatic activity. The products of maltose were strongly attached to the c‐CDots/maltase inhibiting the activity.^[^ ^74^ ^]^

## 5 Summaries and Challenges

In summary, we have discussed the historical evolution of chirality from molecules to nanomaterials (semiconductors), chirality quantification, and the origin of the optical activity of c‐CDots. The synthetic strategies of c‐CDots are also summarized including post‐functionalization of CDots with chiral ligands, one‐pot synthesis, and assembly of CDots with chiral soft templates. The excellent electronic, optical, and biocompatibility properties of c‐CDots have led to numerous applications in diverse research fields. In particular, the chiral effect of c‐CDots on the various applications is closely associated with the conformation‐dependent properties and uses. Despite significant research progress on c‐CDots in recent years regarding their synthesis, chirality origin, properties, and applications, the following three main scientific issues still need to be addressed in order to expand their potential in numerous chiroptical applications.

First of all, it is still extremely challenging to obtain c‐CDots with pure enantiomers exhibiting giant chiroptical responses. For instance, the g‐factors and circularly polarized luminescence intensity of c‐CDots are still weak and the emission wavelength is still uncontrollable. The reported g‐factors of c‐CDots based on CD spectra are only in the order of 10^−3^, while the g‐factors based on CPL spectra are in the order of 10^−2^, which are mostly insufficient for practical applications. Thus, improving the enantiomeric yields of c‐CDots with enhanced g‐factors would offer great optical activities. Improving the g‐factors is critical for using c‐Cdots in the fabrication of chiral LEDs for display applications, an area that is still not explored. The devices based on the CPL of c‐CDots also rely on the fluorescence quantum yield. The low fluorescence quantum yield restricts semiconductor applications. In addition, the CPL spectra of c‐CDots are usually localized at the UV range, emitting blue light. The studies for c‐CDots at long wavelengths, even at the NIR region, are extremely rare. For instance, the water window for the bio‐applications of c‐CDots can be overcome when the emission light is located at NIR regions. Finally, the large‐scale synthesis of c‐CDots with controlled electronic and chiroptical properties is still challenging. By tuning the kinetic and thermodynamic parameters (temperature, time, solvent, pH, nitrogen or sulfur additives, etc) of synthetic processes, the polymerization of carbon cores and chiral additives would be tailored aiming for an enhanced g‐factor and controllable emission wavelength. Finally, dispersing the c‐CDots into the liquid gels would realize the chiroptical response of c‐CDots modified by the surrounded chiral medium.

Second, the chirality origin of c‐CDots is still not well elucidated currently. There are several limitations regarding the experimental and theoretical characterization of c‐Cdots. Generally, c‐CDots are produced by either self‐polymerization or hetero‐polymerization, which does not clearly reveal the electron transition between CDots and chiral stimuli. The whole CDots are composed of organic/inorganic elements, making it difficult to build a theoretical model. Separation and purification of c‐CDots is time‐consuming and needs complicated processing. At the same time, the growth kinetics of c‐CDots are hardly in situ monitored by electron transition microscopy, prohibiting the further study of their inherent structures. It is still unclear whether the inherent structure of the carbon core has a twisted lattice or a non‐twisted lattice since the polymer structures are easily destroyed by the energy of the electron beam in a TEM device. In order to better understand the chiroptical origin of c‐CDots, cryo‐electron microscopy reducing the irradiation damage to the sample would be a better choice for the structural analysis of c‐CDots. Regarding the theoritical simulations, supercomputers could facilitate the simulation of the electron transitions in nanoparticles rather than nanoclusters for better understanding of the mechanism of chiroptical origin. Therefore, the mechanistic understanding of chiral transfer, chirality amplification, and chirality regulation of c‐CDots could be a scientific tool for the guidance of synthesis on a large scale and better design of corresponding applications. Thirdly, to take full advantage of their chiral effects and functionalities, it is important to develop the key applications and technologies of c‐CDots enantiomers so as to reveal the structure‐performance relationship. For instance, it would be interesting to combine other functional domains with c‐CDots in order to extend and modify their chiroptical properties. As an example, when integrating chiral plasmonic metal NPs with c‐CDots, the weak CPL response would be enhanced by the chiral plasmon resonances. At present, there is still a big gap between the synthesis and applications of c‐CDots. AI intelligence based on big data can assist application‐oriented material synthesis. To operate properly and yield meaningful insights and results, AI needs to collect a robust amount of experimental and theoretical data to resume a huge database. Therefore, the use of machine learning to guide the synthesis of c‐Cdots is currently at an early stage, but it has a remarkable growth potential.

## Conflict of Interest

There are no conflicts to declare.
